# Expression of matrix metalloproteinases 1, 3, and 9 in degenerated long head biceps tendon in the presence of rotator cuff tears: an immunohistological study

**DOI:** 10.1186/1471-2474-11-271

**Published:** 2010-11-25

**Authors:** Stefan Lakemeier, Soeren A Schwuchow, Christian D Peterlein, Christian Foelsch, Susanne Fuchs-Winkelmann, Eleni Archontidou-Aprin, Juergen RJ Paletta, Markus D Schofer

**Affiliations:** 1 Department of Orthopedics and Rheumatology, University Hospital Marburg, Marburg, Germany

## Abstract

**Background:**

Long head biceps (LHB) degeneration, in combination with rotator cuff tears, can be a source of chronic shoulder pain. LHB tenotomy reduces pain and improves joint function although the pathophysiological context is not well understood. Tendon integrity depends on the extracellular matrix (ECM), which is regulated by matrix metalloproteinases (MMP). It is unclear which of these enzymes contribute to LHB but we chose to study MMP 1, 3, and 9 and hypothesized that one or more of them may be altered in LHB, whether diagnosed preoperatively or intraoperatively. We compared expression of these MMPs in both LHB and healthy tendon samples.

**Methods:**

LHB samples of 116 patients with degenerative rotator cuff tears were harvested during arthroscopic tenotomy. Patients were assigned to 4 groups (partial thickness tear, full thickness tear, cuff arthropathy, or control) based upon intraoperative findings. Partial and full thickness tears were graded according to Ellman and Bateman's classifications, respectively. MMP expression was determined by immunohistochemistry.

**Results:**

MMP 1 and 9 expression was significantly higher in the presence of rotator cuff tears than in controls whereas MMP 3 expression was significantly decreased. MMP 1 and 9 expression was significantly higher in articular-sided than bursal-sided partial thickness tears. No significant association was found between MMP 1 and 9 expression and full thickness tears, and the extent of the cuff tear by Bateman's classification.

**Conclusion:**

Increased MMP 1 and 9 expression, and decreased MMP 3 expression are found in LHB degeneration. There is a significant association between the size and location of a rotator cuff tear and MMP expression.

## Background

Abnormalities of the long head biceps tendon (LHB) are often associated with rotator cuff tears and may be a reason for persistent shoulder pain [1,2]. Arthroscopic tenotomy of the degenerated LHB usually improves symptoms significantly [3,4]. LHB degeneration can be diagnosed both clinically and radiographically by magnetic resonance imaging (MRI) [5,6]. While tendinopathy has been studied extensively in the supraspinatus, Achilles, patellar, and extensor carpi radialis brevis tendons, there is a paucity of information on LHB tendon degeneration [7-10].

The anatomy of the LHB is unique. The proximal part of the tendon is intraarticular, so pathology is isololated to the biceps tendon itself, or to the glenohumeral joint and surrounding musculature [11]. The extraarticular portion is protected under the pectoralis major, and subjected primarily to tensional strain [12]. Studies on the histopathology of the intraarticular LHB are rare. Longo et al. demonstrated that ruptured tendons exhibit marked histopathologic changes in comparison to cadaveric tendons [13]. However, the molecular basis of tendinopathy is not completely understood.

Much attention has been focused on the matrix metalloproteinases (MMP) in tendinopathy [14,15]. MMPs are a family of 24 zinc-dependent endopeptidases that collectively degrade the extracellular matrix [16]. MMP 1 belongs to the group that cleaves most subtypes of collagen, especially the fibrillar collagens, which provide mechanical strength. MMP 3 is of the stromelysins, broad-spectrum proteinases that also have regulatory functions (such as activation of other MMPs). MMP 9 is a gelatinase, which degrades smaller collagen fragments released during collagenase activity [16]. When comparing the histologic and molecular changes of the intraarticular and extraarticular LHB after tenotomy, Joseph et al. described increased MMP 1 and MMP 3 expression associated with histologic signs of tendinopathy [17].

In our study, we aimed first to demonstrate an alteration of MMP 1, 3, and 9 expression in degenerated LHB compared with healthy controls. Secondly, we hypothesized that there was a correlation between MMP expression in degenerated LHB and the extent of an intraoperatively observed rotator cuff tear.

## Methods

116 patients (55 male, 61 female) were included in our study. Approval was granted by the ethics committee of our institution and informed consent was obtained in all cases. 108 patients had a rotator cuff tear requiring surgery. LHB tissue specimens were harvested from the mid-portion of intraarticular part of LHB by arthroscopic tenotomy during arthroscopic shoulder surgery (performed by MDS). The control group consisted of 8 trauma patients with humeral head fractures. In this group, LHB samples were harvested during humeral head prosthesis implantation. In every control, the rotator cuff was visualized intraoperatively and confirmed to be normal; shoulder osteoarthritis was excluded radiologically.

Patients were divided into four groups, according to the intraoperative findings, as follows: Group I: no shoulder pathology (control group); Group II: partial thickness rotator cuff tear; Group III: full thickness rotator cuff tear; Group IV: cuff arthropathy. Cuff arthropathy was diagnosed during arthroscopy of the shoulder when a massive, irreparable rotator cuff tear combined with complete chondral destruction was found [18]. Partial thickness rotator cuff tears were classified according to Ellman grade (I-III) and were categorized as articular-sided ("A") and bursal-sided ("B") [19]. Full thickness rotator cuff tears were graded according to Bateman's classification (grade I-IV) [20]. Additional file 1 gives a detailed overview of the patient demographics.

### Specimen preparation

LHB samples were immediately fixed in 4% formaldehyde for 24 hours, dehydrated in graded alcohol solution and cedarwood oil, and embedded in paraffin. Sections were cut at 5 μm by a Leica-microtome RM2055 (Leica, Wetzlar, Germany) 40° stainless-steel knife. Masson-Goldner staining was performed (Merck, Darmstadt, Germany) according to the manufacturer instructions.

### Histology

Histomorphometrical analysis was performed at a primary objective lens magnification of 5× fold using a Leica DM5000 microscope and Quips analysis software (Leica) at 40× objective lens magnification. For differentially stained slices, a 10× objective lens magnification was used. Vessel number and size was determined by counting and measuring the vessels in three separate areas in each specimen. Cell counting was performed at a 40× objective lens magnification and recorded by percentage (MMP positive cells per total number of cells per slice).

### Immunochemistry

Slices were rehydrated and incubated in citrate buffer (pH 6) at 97°C for 10 minutes. After blocking with normal serum (horse serum for monoclonal antibodies, goat serum for polyclonal antibodies; Millipore, Billerica, MA, USA), slices were incubated overnight (4°C, humidified chamber) with the antibody against the MMPs (MMP 1: monoclonal antibody, dilution 1:750, MMP 3: polyclonal antibody, dilution 1:100, MMP 9: polyclonal antibody, dilution 1:250).

Immunostaining was performed either using a labeled streptavidin-biotin method (Dako, Hamburg, Germany, REAL Detection System Peroxidase/DAB+), the staining reaction based on 3,3'-diaminobenzidine (DAB) or VECTASTAIN ABC-AP Kit using Vector Red as substrate (Vector laboratories Burlingame, Canada). The stained slices were rinsed with distilled water and stained for 15 seconds with haemalaun as a counterstain. Lastly, sections were rinsed with water and treated with graduated-density alcohol and xylol, as previously described by Matsui et al. in 1998 [21].

### Statistics

Analysis of variance (ANOVA) and modified least square difference (Bonferroni) tests were used for statistical analysis. Data are shown as the mean ± standard error of the mean (SEM). A p-value of < 0.05 was considered statistically significant. The Spearman's rho test was used to evaluate potential correlations.

## Results

### Group I (controls)

**Figure 1:**
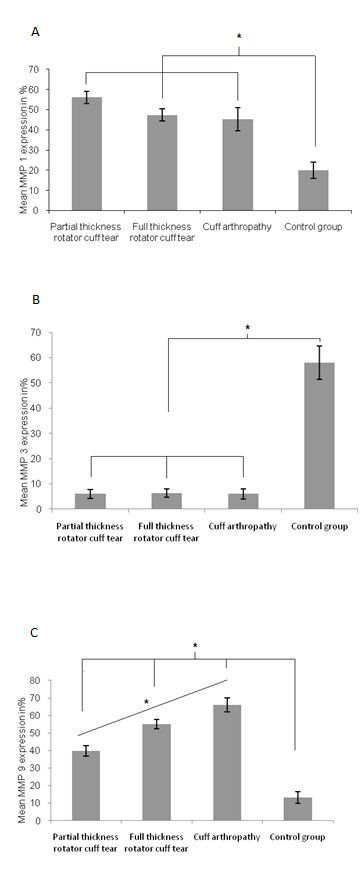
**(a-c): Mean values for percentage of MMP 1 expression (a), MMP 3 expression (b) and MMP 9 expression (c) for the different groups**. Asterisks indicate statistical significant differences.

The control group consisted of 8 patients (4 male, 4 female). The exact values for MMP expression are shown in Additional file 1 and Figure 1a-c.

### Group II (partial thickness rotator cuff tears)

**Figure 2:**
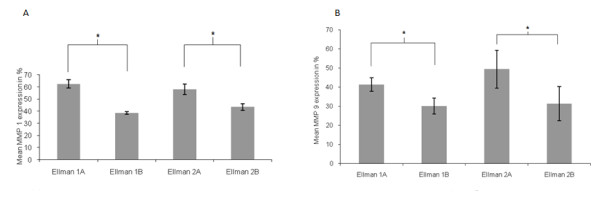
**(a-b): Mean values for percentage of MMP 1 (a) and MMP 9 (b) expression in articular-sided (A) and bursal-sided (B) partial thickness rotator cuff tears grade Ellman I and II**.

There were 48 patients in this group, 33 of which are Ellman grade I. Of these, 28 partial thickness tears were "A" and 5 were "B." 15 patients were Ellman grade II (7 "A," 8 "B"). There were no Ellman III patients. Compared with controls, both MMP 1 and 9 expression was significantly increased whereas MMP 3 expression was significantly decreased (p = 0.027 and 0.035, respectively). Higher levels of MMP 1 and 9 were found in "A" versus "B" partial thickness rotator cuff tears (p = 0.039, Figure 2a-b).

### Group III (full thickness rotator cuff tears)

There were 42 patients in this group. Demographic information, Bateman grades, and MMP 1, 3, and 9 expression are shown in Additional files 1 and 2. MMP 1 expression was significantly higher in this group when compared with controls (p = 0.021). Groups II and IV showed no significant difference in MMP 1 expression. MMP 3 expression was significantly decreased compared to group I (p = 0.012), while no significant difference in MMP 3 expression was seen in Groups II-IV. There was no correlation between MMP 1, 3, and 9 expression and increasing Bateman grade.

### Group IV (cuff arthropathy)

In 18 patients (7 male, 11 female), cuff arthropathy was diagnosed. Patient age was 70 (51-87) years in average. MMP 9 expression was significantly increased in comparison to groups I, II and IV (p = 0.038). MMP 1 expression was significantly higher than in the control group but not significantly augmented in comparison with groups II and III (p = 0.025). MMP 3 expression was significantly decreased compared to the control group (p = 0.043) but there was no significant difference compared to groups II and III.

No statistical correlation could be found between expression of MMP1, MMP 3 and MMP 9 and the age of the 116 included patients.

**Figure 3:**
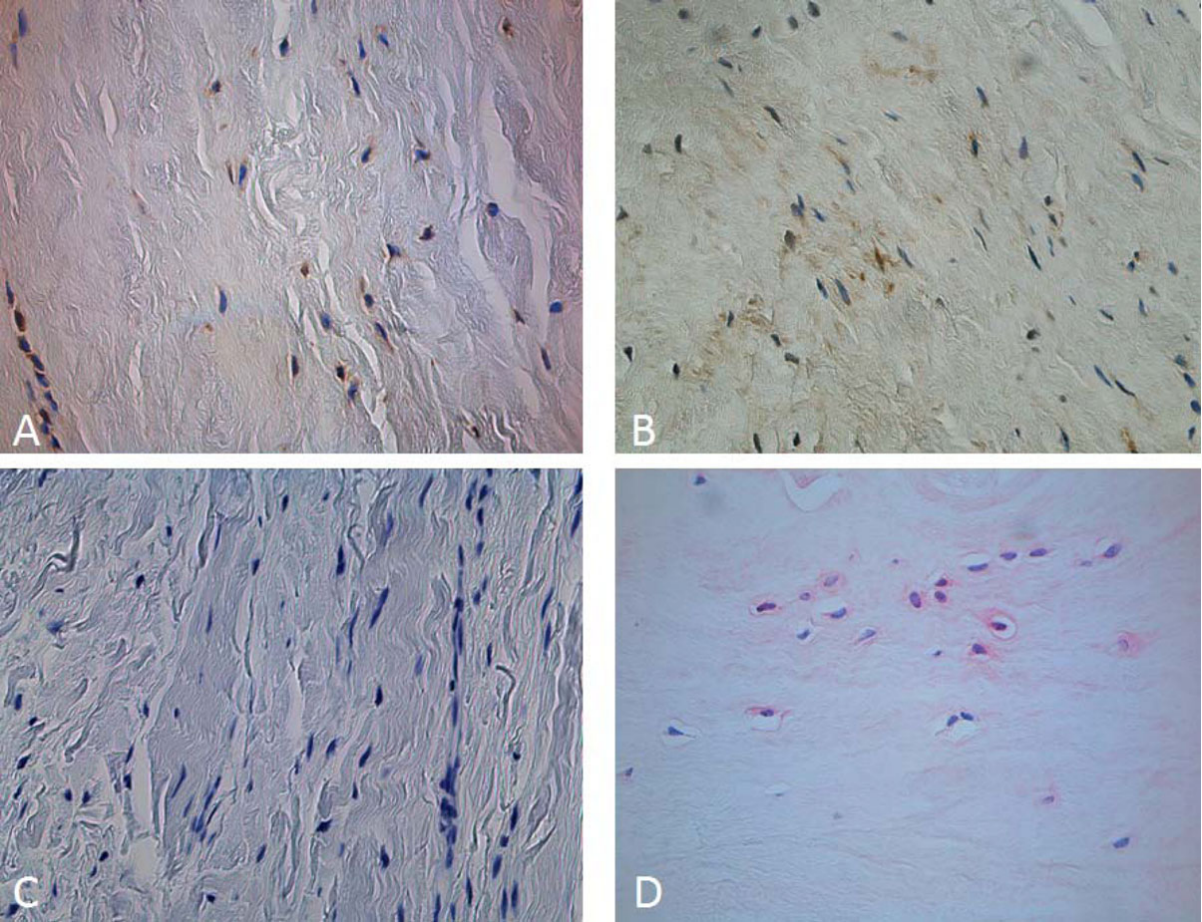
**(a-d): Examples for different stained tendon sections with different MMP antibodies**. Forty-fold objective lens magnification. DAB staining of MMP 1 (a) and MMP 9 (b). Vector red staining of MMP 3 in pathologic (c) and control specimen (d) (40× objective lens magnification).

Examples for stained tendon sections with different antibodies are given in Figure 3(a-d).

## Discussion

We demonstrated that MMP 1 and 9 expression is increased, and MMP 3 expression is decreased in degenerated LHB compared with healthy controls. MMPs play an important role in tendon matrix, as they degrade collagen and proteoglycans in both healthy and diseased patients [22]. Repeated strain is considered to be the major precipitating factor in tendinopathy [23]. MMP 3 has been viewed as a key regulator of extracellular matrix degeneration and remodelling in normal tissue; Jones et al. claimed that its downregulation limits MMP activation within the tissue [24].

Joseph et al. were the first to describe increased levels of MMP 1 and 3 in the intraarticular (compared to the extraarticular) portion of the LHB [17]. In their rabbit flexor tendon model, Asundi and Rempel described MMP 3, but not MMP 1 inhibition [25]. Discrepancies in MMP expression have been noticed when controls were obtained from healthy tissue adjacent to the tendon, or from cadavers. Therefore, caution is required when comparing studies that utilize different types of control tissue [26]. However, multiple authors have confirmed our findings, of increased MMP 1 and 9 as well as decreased MMP 3 expression in degenerated Achilles tendons [26-28]. In this scenario, MMP 1 is considered to be the predominant collagenase [27]. In addition, it has been shown that increased MMP 1 activity occurs in ruptured human supraspinatus tendons [24]. We observed that its activity was highest in group II and decreased with rising extent of shoulder pathology, but this was not statistically significant.

The process of LHB degeneration and its relationship with rotator cuff tears is not well understood. In a cadaveric study comparing 7 shoulders with rotator cuff tears with 7 healthy shoulders, Carpenter et al. could not find any structural or histopathologic differences in the LHB tendon [29]. Thus the authors assumed that LHB retains its material properties in the presence of rotator cuff tears. In contrast to these findings, Peltz et al. demonstrated that LHB mechanical properties worsened over time in a rat model [30]. They assumed that the function of the LHB tendon as a humeral head stabilizer is enhanced when the rotator cuff tendons are weakened; that is, the LHB tendon is required to perform new functions which alters mechanical loading.

Previous histologic studies demonstrated inflammatory infiltrates in degenerated LHB [31,32]. Increased levels of MMP 9 were observed during aseptic tendon inflammation [24]. Karnousou et al. and Olesen et al. showed increased collagen type 1 synthesis in the peritendineum of Achilles tendons after prolonged mechanical loading [33,34]. In vitro, MMP 9 expression can be enhanced by attachment of collagen type 1 [35]. Both findings may explain the presence of enhanced MMP 9 expression due to altered LHB mechanical loading in the presence of rotator cuff tears. Interestingly, in an experimental study on cultured rat Achilles tendon cells published recently, Tsai et al. could reveal an upregulated expression of MMP 1, 8, 9 and 13 after incubation of the cells with ibuprofen a common NSAID that is popular in the treatment of degenerative tendon disease [36]. Likewise, further studies are needed to understand the pathophysiology and the clinical impact of these observations. LHB degeneration may be a result of age-dependent shoulder pathology as it occurs in the presence or absence of cuff tears. Rathbun and Macnab suggested that vascular insufficiency at the entrance to the biceps groove may be responsible for degeneration [37]. These findings are supported by Refior and Sowa who also described LHB degeneration in the bicipital groove of cadavers [11]. However, the fact that MMP 9 expression is enhanced as rotator cuff pathology worsens leads us to assume that LHB occurs secondarily to injury. Our assumption is supported by the work of Peltz et al. and by the fact that full thickness rotator cuff tears are responsible for distinctive glenohumeral instability. In contrast, partial thickness tears cause dynamic instability, especially in mid- and end-range of motion [18,30,38].

In the second portion of our study, we aimed to find a correlation between the extent of rotator cuff tears and MMP expression in the tendon. We demonstrated a significant correlation between the presence of rotator cuff tears and the MMP 1, 3, and 9 expression. In a recent study, Ko et al. found that "A" tears are associated with intrinsic pathologic changes of the shoulder joint, while "B" tears are found in impingement syndrome, a milder underlying condition [39]. Our findings confirmed these observations, in that MMP 1 and 9 expression was significantly higher in "A" versus "B" tears. Hence, it appears that LHB degeneration may be secondary to intraarticular pathological changes. Unfortunately, we did not have any patients with Ellman grade III tears to test this hypothesis. Biomechanical research should be applied to elucidate this finding.

The reason for the development of rotator cuff tears is still controversial. Some authors argue that cuff tears develop from mechanical damage of the tendon caused by subacromial impingement [40]. Others claim that degeneration is due to partial tendon hypovascularisation or primary tendon degeneration [41]. The only consensus is that partial thickness tears may develop into full thickness tears. Gohlke showed that degenerative full thickness rotator cuff tears were more likely to occur in older versus younger patients, a finding we confirmed in our study [42]. In addition, a full thickness tear may progress to cuff arthropathy [43]. We showed that MMP 9 expression increased with the degree of the tear and was highest in group IV. Therefore, our findings suggest that LHB degeneration is related to the course of degenerative rotator cuff disease. While LHB degeneration and degenerative shoulder disease may develop concomitantly due to common etiological factors, we believe that LHB degeneration follows degenerative shoulder disease. This has not been previously reported and further research is necessary for this association to be completely understood.

Full thickness rotator cuff tears may develop into cuff arthropathy, so that many full thickness rotator cuff tears are associated with osteoarthritis of the shoulder. Osteoarthritis was not measured but might influence MMP expression. Furthermore, every patient in group IV had a massive rotator cuff tear, making it difficult to differentiate between the two groups. This bias could be balanced by the high density of patients in each group.

Our study has several limitations. Our control group is small in comparison to the other groups. As MMPs play a pivotal role in the formation of certain tumors, we had to exclude tumor patients who needed prosthesis implantation or upper limb amputation. We chose to obtain LHB samples of trauma patients with comminuted humeral head fractures who required humeral head prosthesis implantation. This indication is rare in young patients and the operation is usually performed in the elderly. We were able to include 8 patients with healthy LHB tendon and rotator cuffs as controls. Although we attempted to find controls that were matched for age with the other groups, trauma patients are younger on average. If LHB degeneration was a normal result of aging, the age factor could partially explain our results. As there was no significant statistical correlation between age and MMP 1, 3, and 9 expression, we do not think that would explain our findings. Furthermore, the difference in age is relatively small between groups.

We demonstrated that MMP 1 and 9 expression is increased, and MMP 3 expression is decreased in degenerated LHB compared with healthy controls. We also showed a significant correlation between the presence of rotator cuff tears and the MMP 1, 3, and 9 expression. We also believe that LHB degeneration follows degenerative shoulder disease, a claim that has not previously been reported. Further research is necessary to clarify the role of the MMPs in the course of degenerative LHB and rotator cuff disease.

## Conclusion

LHB degeneration is associated with increased MMP 1 and 9 expression and decreased MMP 3 expression. It appears that LHB degeneration is secondary to the development of rotator cuff tears and is aggravated over the course of degenerative shoulder disease.

## Competing interests

The authors declare that they have no competing interests.

## Authors' contributions

SL was the main composer of the manuscript. SAS, EAA and CF performed histologic and immunohistologic testing. JRJP was involved in the study design, the immunohistologic examinations, and statistical analyses. SFW conceived the study and participated in its design and coordination. CDP participated in the coordination of the study and helped to draft the manuscript. MDS designed the study, performed the surgeries, and obtained LHB samples. All authors read and approved the final manuscript.

## Pre-publication history

The pre-publication history for this paper can be accessed here:

http://www.biomedcentral.com/1471-2474/11/271/prepub

## Supplementary Material

Additional file 1 **Overview of patient demographics**. Presentation of shoulder pathology classification and mean MMP 1, 3, and 9 expression among different groups. Click here for file

Additional file 2 **Overview of mean MMP 1, 3 and 9 expression**. Mean MMP 1, 3, and 9 expression for the different grades of full thickness rotator cuff tears. Click here for file
