# Supplementary material for: Expression of matrix metalloproteinases 1, 3, and 9 in degenerated long head biceps tendon in the presence of rotator cuff tears: an immunohistological study
Source: BMC Musculoskelet Disord. 2010 Nov 25;11:271. doi: 10.1186/1471-2474-11-271 (PMC2998463; doi:10.1186/1471-2474-11-271)
Supplement: Additional file 1 — Overview of patient demographics. Presentation of shoulder pathology classification and mean MMP 1, 3, and 9 expression among different groups. [file 1471-2474-11-271-S1.DOC]

| **Diagnosis** | **Number of patients** | **Number of patients male** | **Number of patients female** | **Mean age**  **(years)** | **MMP 1 expression in % ± standard error** | **MMP 3 expression in % ± standard error** | **MMP 9 expression in % ± standard error** |
| --- | --- | --- | --- | --- | --- | --- | --- |
| **Control group (group I)** | 8 | 4 | 4 | 56 (37-69) | 20.00±3.92 | 58.00±6.55 | 13.33±3.33 |
| **Partial thickness rotator cuff tear (group II)** | 48 | 24 | 24 | 61 (39-78) | 56.07±3.05 | 6.00±1.88 | 39.76±2.98 |
| **Full thickness rotator cuff tear (group III)** | 42 | 20 | 22 | 67 (55-80) | 47.31±3.05 | 6.29±1.62 | 55.11±2.63 |
| **Cuff arthropathy** **(group IV)** | 18 | 7 | 11 | 70 (51-87) | 45.21±5.76 | 6.00±1.91 | 66.02±4.02 |

Additional file 1:

Title: Overview of patient demographics.

Description: Presentation of shoulder pathology classification and mean MMP 1, 3, and 9 expression among different groups.
