# Supplementary material for: Expression of matrix metalloproteinases 1, 3, and 9 in degenerated long head biceps tendon in the presence of rotator cuff tears: an immunohistological study
Source: BMC Musculoskelet Disord. 2010 Nov 25;11:271. doi: 10.1186/1471-2474-11-271 (PMC2998463; doi:10.1186/1471-2474-11-271)
Supplement: Additional file 2 — Overview of mean MMP 1, 3 and 9 expression. Mean MMP 1, 3, and 9 expression for the different grades of full thickness rotator cuff tears. [file 1471-2474-11-271-S2.DOC]

| **Grade Bateman classification** | **Bateman I** | **Bateman II** | **Bateman III** | **Bateman IV** |
| --- | --- | --- | --- | --- |
| **Number of patients** | 3 | 15 | 11 | 13 |
| **Mean MMP 1 expression in % ± standard error** | 36.00±9.50 | 55.00±5.24 | 41.34±5.33 | 52.00±4.66 |
| **Mean MMP 3 expression in % ± standard error** | 3.00±0.55 | 9.57±1.35 | 1.33±0.39 | 4.57±1.20 |
| **Mean MMP 9 expression in % ± standard error** | 51.00±2.18 | 56.63±4.44 | 56.00±3.99 | 52.33±3.34 |

Additional file 2:

Title: Overview of mean MMP 1, 3 and 9 expression

Description: Mean MMP 1, 3, and 9 expression for the different grades of full thickness rotator cuff tears.
